# CHILDREN’S HEALTH: Secondhand Smoke Exposure May Alter Fetal Blood Pressure Programming

**DOI:** 10.1289/ehp.118-a158a

**Published:** 2010-04

**Authors:** Adrian Burton

**Affiliations:** **Adrian Burton** is a biologist living in Spain who also writes regularly for *The Lancet Oncology*, *The Lancet Neurology*, and *Frontiers in Ecology and the Environment*

Babies born to mothers who smoke cigarettes may be at risk for abnormal blood pressure and heart rate control at birth, suggests research published in the March 2010 issue of *Hypertension*. The results of the new study further hint that this control may become worse as exposure to secondhand smoke continues, perhaps increasing the risk of developing hypertension in later life.

The study compared the heart rate and blood pressure control of 19 infants born to nonsmokers with those of 17 infants whose mothers reported smoking an average of 15 cigarettes per day before and after giving birth. The resting blood pressure of the infants in both groups followed essentially the same developmental trend over the first year of life, although the smoke-exposed infants had higher diastolic blood pressure at age 3 months. The resting heart rate of both groups also was similar and followed the same trend up to age 3 months. But by 1 year the resting heart rate of the smoke-exposed infants averaged 20% slower than that of their unexposed counterparts.

The researchers also monitored changes in heart rate and blood pressure over a span of 40 beats as the infants, sleeping soundly on tilt tables, were raised from a supine position to an inclination of 60º over 5 seconds and held in that position for 1 minute. “As the body becomes more upright, the heart rate should rise temporarily, and different blood vessels should constrict to increase the blood pressure and ensure enough blood gets to the brain,” explains first author Gary Cohen, a senior research scientist in the Department of Women’s and Children’s Health at the Karolinska Institute, Stockholm. Sure enough, that is what the authors observed for the nonexposed infants, with peak values becoming somewhat higher between 1 week and 1 year as expected.

The exposed infants showed a similar trend over time. However, between 3 months and 1 year their responses became exaggerated, with their heart rate rising faster (and increasing by an average of 11.5% instead of the 6.5% seen in the nonexposed babies) before falling more quickly. Their diastolic blood pressure followed suit.

When the nonexposed infants were tilted and maintained upright, sustained rises in systolic, diastolic, and mean blood pressure of 2–3% were seen at age 1 week, rising to 8–10% by 1 year as expected. In contrast, in the exposed infants the increases in blood pressure were nearly double at age 1 week but failed to increase over time.

“Thus, the newborns of smokers hyperreact to positional change, but by the time they are one year old and want to stand up they are underreacting; their routine blood pressure compensation systems just don’t work properly,” says Cohen. “It would appear that neither their heart rate nor sympathetic constrictor tone [impulses from the sympathetic nervous system that help control blood vessel constriction] are properly ‘programmed’ even at birth, with things getting worse over time.”

This programming problem could lie in an overly strong sympathetic tone caused by exposure to some compound in cigarette smoke in the womb and after birth, the researchers say. This might slowly increase vascular resistance, leading to the increased diastolic blood pressure seen at rest at 3 months, and the eventual loss of sympathetic reactivity.

The authors further hypothesize that the fall in heart rate observed in exposed infants at age 1 year was an attempt to restore some kind of equilibrium. Unfortunately, this reprogramming solution appears to hinder proper positional blood pressure control, “and there is evidence this could increase the chances of hypertension later on,” explains Cohen.

In adults, cardiovascular pathophysiology can involve chronic sympathetic overactivity leading to increased blood pressure. The authors suggest something similar may be happening in the children they studied.

“[Whether these observations can be] explained by alterations in central sympathetic outflow requires further investigation as this was not directly assessed in this study,” remarks James Fisher, a lecturer in exercise physiology in the School of Sport and Exercise Sciences, University of Birmingham who was not involved in the study. “As is often the case with good research, we are left with more questions than answers. Is the altered cardiovascular reactivity specific to postural stress, or is it more generalized? What is the biological significance of the magnitude of the alteration in cardiovascular reactivity? How permanent is the ‘reprogramming,’ and is it reversible if smoke exposure is withdrawn?”

Although interesting, the study is rather small. “I would like to see confirmation in a larger study,” says Mark Caulfield, director of the William Harvey Research Institute at Barts and The London School of Medicine and Dentistry, “with formal proof of cigarette consumption status in each of the study groups before drawing a firm conclusion.”

## Figures and Tables

**Figure:**
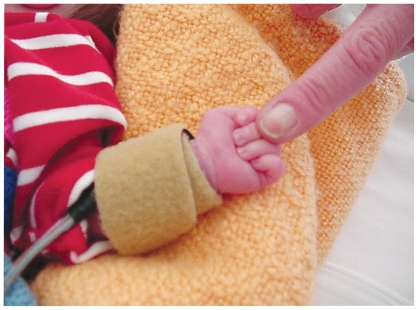
Prenatal exposure to maternal smoking may have contributed to blood pressure abnormalities observed in infants.

